# Effects of the n-6/n-3 polyunsaturated fatty acids ratio on postprandial metabolism in hypertriacylglycerolemia patients

**DOI:** 10.1186/1476-511X-12-181

**Published:** 2013-12-10

**Authors:** Zhixiu Song, Ligang Yang, Guofang Shu, Huixia Lu, Guiju Sun

**Affiliations:** 1 Key Laboratory of Environmental Medicine and Engineering of Ministry of Education, and Department of Nutrition and Food Hygiene, School of Public Health, Southeast University, 87 Ding Jia Qiao Road, Nanjing 210009, China; 2 Second Clinical Medical College, Nanjing University of Traditional Chinese Medicine, 138 Xian Lin Road, Nanjing 210046, China; 3 Zhongda Hospital Affiliated, Southeast University, 87 Ding Jia Qiao Road, Nanjing 210009, China

**Keywords:** n-6 PUFAs, n-3 PUFAs, Postprandial metabolism, Hypertriacylglycerolemia, Inflammatory, Endothelial function

## Abstract

**Background:**

Atherosclerosis is a postprandial phenomenon. The balanced n-6/n-3 PUFA ratio contributing to the prevention of atherosclerosis has been well shown, but the effect of the ratio on postprandial metabolism has not been fully investigated. The aim of this study was to investigate the effects of the n-6/n-3 PUFAs ratio on postprandial metabolism in hypertriacylglycerolemia patients, comparing them to healthy controls.

**Methods:**

Test meals with 0.97 (high n-3) and 8.80 (low n-3) n-6/n-3 PUFAs ratio were administered in a randomized crossover design to 8 healthy and 8 hypertriacylglycerolemia subjects. Blood samples were collected for 8 hours after meals to measure triglyceride (TG), total cholesterol (TC), HDL, ApoA, ApoB, glucose, insulin, inflammatory makers including tumor necrosis factor alpha (TNF-α) and interleukin-6 (IL-6), endothelial function including nitric oxide (NO) and endothelin-1 (ET-1).

**Results:**

According to repeated–measures ANOVA, the postprandial response of lipid, glucose, insulin, inflammation and endothelial function were not significantly different between meals. The postprandial TG and NO response were significantly different between healthy control (HC) and hypertriglyceridemia group (HTG) after both meals (P < 0.01). After both meals maximal change and iAUC for TG was all higher in HTG group than HC group, the difference was significant after low n-3 meal but not after high n-3 meal. The concentration of glucose, insulin, IL-6, TNFα and ET-1 at each time point was higher and NO was lower in HTG group, but the maximal change and iAUC had no significant difference except for iAUC of insulin, IL-6 and diAUC of NO after low n-3 meal.

**Conclusions:**

The ratio of n-6 and n-3 maybe do not acutely influence the postprandial metabolism, inflammatory response and endothelial function, but the low n-3 meal can strengthen the difference between HTG and HC group.

## Background

Most of the time is spent in not-fasting state for most people consuming meals at regular 4-5 h. Since Zilversmit first suggested that the postprandial lipemia was linked with atherosclerosis [1], postprandial metabolism had been received much more attention. The exacerbated postprandial response characterizes with retarded clearance of postprandial triglyceride-rich lipoproteins (TRLs), which caused in part by the increasing of triglycerides (TG) in average, peak and later level after a fat meal [2,3]. Delayed clearance of TRLs can cause the increasing of inflammation makers and impairment of endothelial function, which promote the formation and development of atherosclerosis [4,5]. The magnitude of the postprandial response is determined by several factors such as quality and quantity of meal intake, characteristics of the subjects, lifestyle and habitual dietary composition [2]. It is worthy noting that postprandial response can be influenced by the amount and type of dietary fatty acids presented in the test meal.

There are three categories of fatty acids: saturated fatty acids (SFAs), monounsaturated fatty acids (MUFAs) and polyunsaturated fatty acids (PUFAs). Studies have revealed important differences with postprandial lipid responses being of the order SFAs>MUFAs>PUFAs [6-8]. The n-3 and n-6 series PUFAs compete with each other for enzymes required when they are synthesized and all play an important role in vivo [9,10]. The n-3 fatty acids-derived eicosanoids are anti-inflammatory, whereas those formed from n-6 fatty acids are pro-inflammatory [11]. So a balanced n-6/n-3 ratio contributed to the prevention of many inflammatory related diseases such as atherosclerosis [12,13]. Several sources of information suggest that the present diet is deficient in n-3 fatty acids with a ratio of n-6 to n-3 of about 10:1 [14,15]. Many researches showed that the high ratio of n-6 and n-3 can increase the fasting concentrations of TG [16-18] and inflammatory markers [19] , but whether the test meal with low ratio of n-6 and n-3 acutely improving postprandial lipid, glucose and inflammatory response, whether ameliorating the postprandial endothelial disfunction have not been thorough investigated.

Therefore we hypothesized that the low n-6/n-3 ratio of the test meal could modulate postprandial response and the effect would be more pronounced in hypertriacylglycerolemia subjects. We investigated the metabolic response to high fat meals only differing the ratio of n-6 and n-3 (high n-3 or low n-3), in hypertriacylglycerolemia and healthy subjects. The study will provide the theoretical and practical basis for recommending the optimal ratio of n-6 to n-3 and for prevention and treatment of hyperlipemia and metabolism disorder, and then for prevention of the development of atherosclerosis in human being.

## Methods

### Subjects

The sample was composed by 16 adults recruited from the local community. The subjects were classified into two groups based on the fasting blood triglycerides level: hypertriglyceridemia group (HTG group, n = 8) and healthy control group (HC group, n = 8). Subjects were excluded if they had diabetes, coronary heart disease, hyperthyroidism, malignant tumor and other chronic inflammatory diseases, taking drugs of lipid-lowering, anti-inflammatory and affecting lipid metabolism in the past one month, taken n-3PUFAs supplements in the past 6 months, or had any disease or condition known to affect study end points. The baseline characteristics of subjects are summarized in Table 1.

**Table 1:** The baseline characteristics of subjects

	**HC group**	**HTG group**	**P**
	**(n = 8)**	**(n = 8)**	
Sex (male/femal)	8(4/4)	8(4/4)	NS
Age (years)	45.8 ± 9.41	52.8 ± 9.28	NS
Height (m)	1.69 ± 0.09	1.68 ± 0.07	NS
Body mass (kg)	65.40 ± 8.78	71.33 ± 9.99	NS
BMI (kg · m^−2^)	22.87 ± 1.99	25.08 ± 2.69	NS
Waist-to-hipratio(WHR)	0.87 ± 0.05	0.94 ± 0.49	0.036
Systolic blood pressure (mm Hg)	75.00 ± 9.27	85.33 ± 5.16	NS
Diastolic blood pressure (mm Hg)	119.50 ± 11.34	131.00 ± 16.33	NS

The study was approved by the ethic committee of Zhongda hospital affiliated Southeast University, and had been registered on Chinese clinical trial registry (2012ZD11KY17.0). All subjects were informed about the study process, probable problem and their rights and signed their written informed consent before they entered the screening procedure.

### Study design

The study used a cross-over randomized controlled design and consisted of 2 d oral lipid tolerance test, separated by a washout period of at least 2 weeks. During the study period subjects were required to maintain their usual lifestyle and diet habits. On the day prior to the test day, subjects were asked to refrain from alcohol, high-fat food and strenuous exercise. After a 10-h overnight fast, an intravenous catheter was inserted into a forearm vein for collecting blood samples. After taking a fasting blood sample, each participant was requested to consume one of the test meals (18 kcal energy per kilogram body weight). Subsequent blood samples were collected at 30 min, 1 h, 2 h, 4 h, 6 h, 8 h after meal consumption. The serum was separated by centrifugation at 3000 g for 20 min then stored at −80°C until analyzed.

To eliminate the differences in metabolism, the liquid test meals were prepared. The test meals were isoenergetic and all consisted of casein, edible oil (butter, corn oil, linseed oil and olive oil), sugar, lactose, malt dextrin, monoglycerides and water with a caloric distribution of 60% from fat, 15% from protein and 25% from carbohydrates. The compositions of the two kind meals were the same except of the different in n-6/n-3 ratio while maintaining a PUFAs/MUFAs/SFAs ratio of approximately 1/1/1 because of the edible oil composition. The food content and composition of the test meals are listed in Table 2.

**Table 2:** The food content and composition of the test meals

		**High n-3**	**Low n-3**
Food content/1000 kcal			
Casein(g)		37.50	
Sugar(g)		39.38	
Malt dextrin(g)		18.75	
Lactose(g)		4.38	
Edible oil	Butter(g)	23.02	21.54
	Corn oil(g)	12.81	35.86
	Linseed oil(g)	19.87	2.99
	Olive oil(g)	10.96	6.28
Composition			
Energy (kcal)		1000 kcal	
Protein (% of energy)		15%	
Carbohydrates (% of energy)		25%	
Fat (% of energy)		60%	
SFA:MUFA:PUFA		1.00:1.06:1.10	1.00:1.09:1.13
n-6/n-3 ratio		0.97	8.80

### Laboratory assessments

Concentrations of TG, TC, HDL, ApoA, ApoB, glucose, insulin, TNFα and IL-6 were determined in serum samples from T = 0, 0.5, 1, 2, 4, 6 and 8 h after meal consumption. ET-1 and NO concentrations were measured from T = 0, 2, 4, 6 and 8 h after meal consumption. TG, TC and HDL concentrations were determined by enzymatic assays. Glucose concentrations were determined by the glucose oxidase method. Insulin was quantified by chemical immune assay. The concentrations of ApoA and ApoB were detected by immune turbidimetric method. Concentrations of TNFα, IL-6, and ET-1 were measured using ELISA kits purchased from Science Biotechnology Co. Ltd. (Yantai, China). Concentrations of NO were analyzed using Griess method and the assay kit was obtained from Applygen Technologies (Beijing, China).

### Statistical analysis

Data were expressed as mean ± SD for normally distributed. The incremental areas under the postprandial curve (iAUC) or the decremental AUC (diAUC) and maximal change were used to evaluate the overall response during postprandial period. AUC was calculated using GraphPad Prism4.03. Maximal change was calculated by subtracting fasting concentrations from maximal value or by subtracting minimal value from fasting concentrations. Differences in AUC and maximal change between the test meals and subject groups were tested for significance by univariate independent-sample T test. Analysis of variance (ANOVA) for repeated measures was used to analyze the time and meals interaction within subject groups and the time and group interaction within meals. All these analyses were performed using SPSS 17.0. Values with P < 0.05 were considered statistically significant in all cases.

## Results

### TG

**Figure 1:**
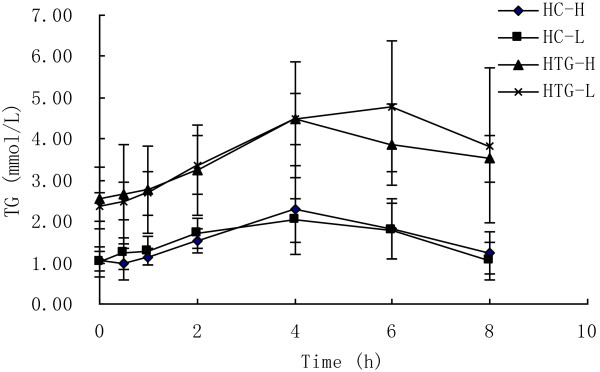
**TG concentrations over 8 h after high n-3 and low n-3 in HC and HTG group.** Data presented as mean ± SD (n = 8). TG concentrations increased significantly after both meals in both groups (P<0.01). The postprandial response of TG concentration was not significantly different between the two meals in both groups, but was significantly different between HC group and HTG group after the two meals.

As showed in Figure 1, there was a significant change in TG concentration over time in both groups after both meals (P < 0.01). In HC group the concentrations of TG reached peak concentrations after 4 h and had returned to fasting concentrations after 8 h after both meals. But in HTG group the concentrations of TG reached peak concentrations after 4 h after high n-3 meals but after 6 h after low n-3 meals, and had not returned to fasting concentrations after 8 h after both meals. The postprandial response of TG concentration was not significantly different between the two meals in HC group (P = 0.797) and HTG group (P = 0.975), however was significantly different between HC group and HTG group after the two meals (P < 0.01). The maximal change and iAUC in TG was lower after high n-3 meals than after low n-3 meals in HTG groups, but there was not significant difference between two meals in both groups. The maximal change and iAUC in TG was lower in HC group than in HTG group after both meals, and there was significant difference between two groups after low n-3 (Table 3).

**Table 3:** Maximal change and AUC from fasting concentrations (mean ± SD)

	**HC group**		**HTG group**		**P for meals effect**		**P for group effect**	
	**High n-3**	**Low n-3**	**High n-3**	**Low n-3**	**HC group**	**HTG group**	**High n-3**	**Low n-3**
TG								
Maximal change	1.27 ± 0.91	1.04 ± 0.33	1.95 ± 0.61	2.80 ± 1.29	0.586	0.177	0.16	0.009
iAUC	5.09 ± 3.30	5.02 ± 1.64	8.78 ± 4.11	12.53 ± 5.88	0.966	0.229	0.117	0.013
Glucose								
Maximal change	1.41 ± 0.70	1.47 ± 0.63	2.53 ± 1.31	2.41 ± 1.26	0.89	0.91	0.25	0.132
iAUC	2.73 ± 2.30	1.59 ± 0.87	6.41 ± 5.71	5.13 ± 3.02	0.296	0.638	0.175	0.02
Insulin								
Maximal change	28.36 ± 13.97	37.33 ± 30.68	54.68 ± 42.71	70.15 ± 44.61	0.529	0.558	0.198	0.168
iAUC	61.17 ± 39.01	72.36 ± 56.30	128.47 ± 96.69	160.38 ± 88.92	0.697	0.565	0.161	0.068
IL-6								
Maximal change	65.93 ± 11.56	90.59 ± 53.60	90.88 ± 51.07	126.44 ± 23.11	0.317	0.151	0.291	0.325
iAUC	336.17 ± 140.69	370.57 ± 115.63	466.55 ± 274.55	569.25 ± 177.11	0.653	0.459	0.163	0.044
TNFα								
Maximal change	101.07 ± 33.86	118.96 ± 26.17	119.40 ± 39.41	151.52 ± 91.31	0.069	0.386	0.064	0.289
iAUC	446.41 ± 99.53	573.68 ± 115.90	619.93 ± 178.69	831.04 ± 525.05	0.332	0.456	0.408	0.434
ET-1								
Maximal change	76.02 ± 59.68	79.11 ± 38.90	72.99 ± 42.55	85.44 ± 55.87	0.918	0.673	0.921	0.824
iAUC	314.50 ± 221.10	387.82 ± 180.64	292.71 ± 175.27	371.33 ± 281.99	0.543	0.575	0.854	0.906
NO								
Maximal change	127.64 ± 124.88	125.11 ± 58.39	76.01 ± 69.46	107.62 ± 62.99	0.965	0.428	0.397	0.629
diAUC	575.30 ± 355.16	711.93 ± 302.45	276.00 ± 130.74	339.57 ± 161.64	0.49	0.471	0.081	0.024

### Glucose and insulin

**Figure 2:**
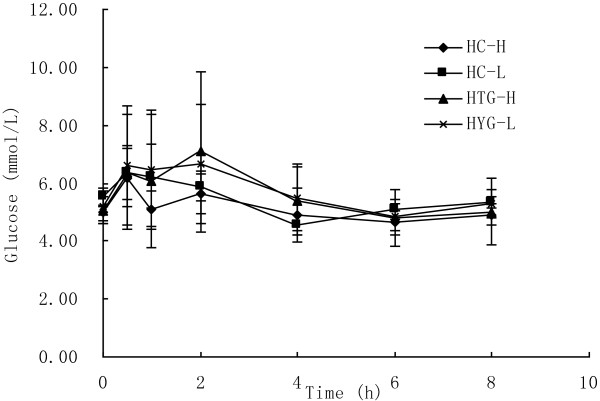
**Glucose concentrations over 8 h after high n-3 and low n-3 in HC and HTG group.** Data presented as mean ± SD (n = 8). Glucose concentrations increased significantly after both meals in both groups (P<0.01). The postprandial response of glucose concentration was not significantly different between the two meals in both groups, and not significantly different between HC group and HTG group after the two meals.

As showed in Figure 2, glucose concentration changed significantly over time in both groups after both meals (P < 0.05). In HC group the concentrations of glucose reached peak concentrations after 0.5 h and had returned to fasting concentrations after 4 h after both meals. But in HTG group the concentrations of glucose reached peak concentrations after 2 h and had returned to fasting concentrations after 6 h after both meals. The postprandial response of glucose concentration was not significantly different between the two meals in HC group (P = 0.057) and HTG group (P = 0.873), and was not significantly different between HC group and HTG group after high n-3 meals (P = 0.346) and low n-3 meals (P = 0.614). The iAUC of glucose was higher after high n-3 meals than after low n-3 meals in both groups, but there was not significant difference. The maximal change and the iAUC in glucose was lower in HC group than in HTG group after both meals, and the iAUC was significantly different between two groups after low n-3 (Table 3).

**Figure 3:**
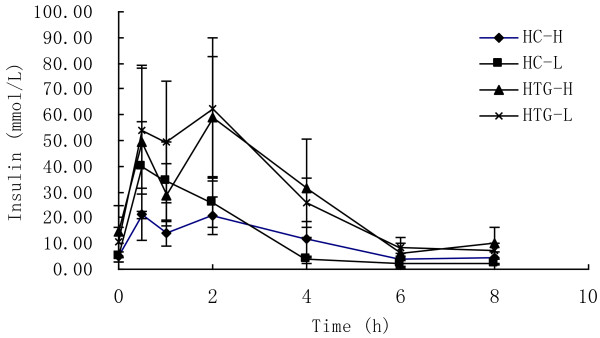
**Insulin concentrations over 8 h after high n-3 and low n-3 in HC and HTG group.** Data presented as mean ± SD (n = 8). Insulin concentrations changed significantly over time after both meals in both groups (P<0.01). The postprandial response of glucose concentration was not significantly different between the two meals in both groups, and was not significantly different between HC group and HTG group after the two meals.

As showed in Figure 3, insulin concentration changed significantly over time in both groups after both meals (P < 0.01). In HC group the concentration of insulin reached peak concentrations after 0.5 h, while in HTG group the concentrations of insulin reached peak after 2 h. The postprandial response of insulin concentration was not significantly different between the two meals in HC group (P = 0.429) and HTG group (P = 0.864), and was not significantly different between HC group and HTG group after high n-3 meals (P = 0.153) and low n-3 meals (P = 0.189). The maximal change and iAUC in insulin was lower after high n-3 meals than after low n-3 meals in both groups, lower in HC group than in HTG group after both meals, but there was all not significant difference (Table 3).

### Inflammatory markers

**Figure 4:**
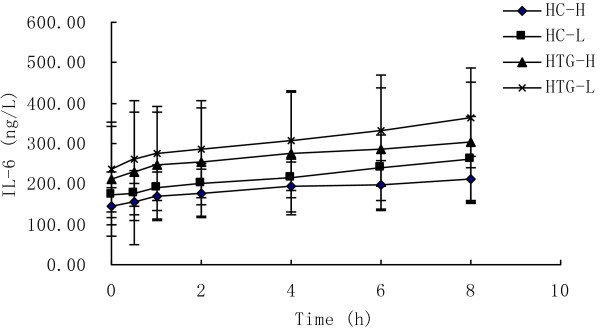
**IL-6 concentrations over 8 h after high n-3 and low n-3 in HC and HTG group.** Data presented as mean ± SD (n = 8). IL-6 concentrations changed significantly over time after both meals in both groups (P<0.05). The postprandial response of IL-6 concentration was not significantly different between the two meals in both groups, and was not significantly different between HC group and HTG group after the two meals.

As showed in Figure 4, IL-6 concentration increased significantly from baseline in both groups after both meals (P < 0.05). At each time point after both meals the concentrations of IL-6 in HTG group were higher than in HC group. The postprandial response of IL-6 concentration was not significantly different between the two meals in HC group (P = 0.421) and HTG group (P = 0.635), and was not significantly different between HC group and HTG group after high n-3 meals (P = 0.233) and low n-3 meals (P = 0.149). The maximal change and the iAUC in IL-6 were lower after high n-3 meals than after low n-3 meals in both groups, but there was not significant difference. The maximal change and the iAUC in IL-6 were lower in HC group than in HTG group after both meals, and the iAUC was significantly different between two groups after low n-3 (Table 3).

**Figure 5:**
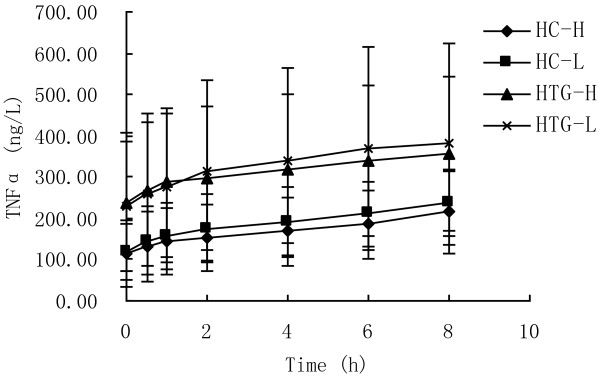
**TNFα concentrations over 8 h after high n-3 and low n-3 in HC and HTG group.** Data presented as mean ± SD (n = 8). TNFα concentrations changed significantly over time after both meals in both groups (P<0.05). The postprandial response of TNFα concentration was not significantly different between the two meals in both groups, and was not significantly different between HC group and HTG group after the two meals.

As showed in Figure 5, TNF-α concentration increased significantly from baseline in both groups after both meals (P < 0.05). The postprandial response of TNFα concentration was not significantly different between the two meals in HC group (P = 0.712) and HTG group (P = 0.943), and was not significantly different between HC group and HTG group after high n-3 meals (P = 0.107) and low n-3 meals (P = 0.170). At each time point after both meals the concentrations of TNFα in HTG group were higher than in HC group. The maximal change and iAUC in TNFα was lower after high n-3 meals than after low n-3 meals in both groups, lower in HC group than in HTG group after both meals, but there was all not significant difference (Table 3).

### Endothelial function

**Figure 6:**
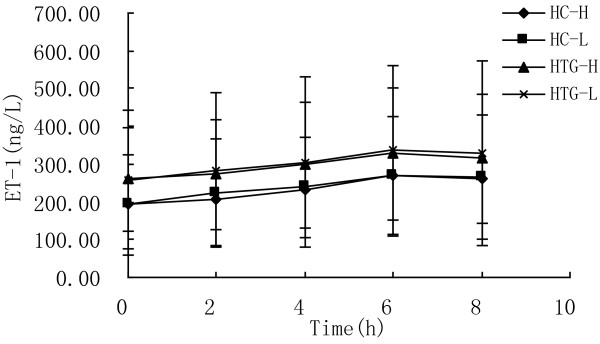
**ET-1 concentrations over 8 h after high n-3 and low n-3 in HC and HTG group.** Data presented as mean ± SD (n = 8). ET-1 concentrations changed significantly over time after both meals in both groups (P<0.05). The postprandial response of ET-1 concentration was not significantly different between the two meals in both groups, and was not significantly different between HC group and HTG group after the two meals.

As showed in Figure 6, ET-1 concentration increased significantly from baseline in both groups after both meals (P < 0.05). At each time point after both meals the concentrations of ET-1 in HTG group were higher than in HC group. The postprandial response of ET-1 concentration was not significantly different between the two meals in HC group (P = 0.959) and HTG group (P = 0.941), and was not significantly different between HC group and HTG group after high n-3 meals (P = 0.504) and low n-3 meals (P = 0.557). The maximal change and iAUC in ET-1 was lower after high n-3 meals than after low n-3 meals in both groups, but there was not significant difference (Table 3).

**Figure 7:**
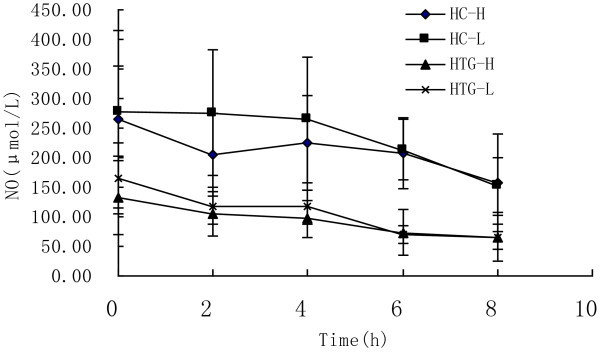
**NO concentrations over 8 h after high n-3 and low n-3 in HC and HTG group.** Data presented as mean ± SD (n = 8). NO concentrations changed significantly over time after both meals in both groups (P<0.01). The postprandial response of NO concentration was not significantly different between the two meals in both groups, and was significantly different between HC group and HTG group after the two meals.

As showed in Figure 7, NO concentration decreased significantly from baseline in both groups after both meals (P < 0.05). At each time point after both meals the concentrations of NO in HTG group were lower than in HC group. The postprandial response of NO concentration was not significantly different between the two meals in HC group (P = 0.522) and HTG group (P = 0.402), and was significantly different between HC group and HTG group after both test meals (P < 0.01). The maximal change and the diAUC in NO were higher in HC group than in HTG group after both meals, and the diAUC was significantly different between two groups after low n-3 (Table 3).

## Discussion

To our knowledge, the current research is the first time to investigate the effect of the n-6/n-3 PUFAs ratio on the postprandial lipid, glucose and insulin metabolism, inflammatory response and endothelial function in hypertriacylglycerolemia subjects, comparing them to normal controls. According to repeated–measures ANOVA, the postprandial response of lipid, glucose, insulin inflammation and endothelial function after high n-3 test meal were not significantly different from that after low n-3 test meal, but the postprandial lipid response was exaggerated in HTG subjects regardless of test meal, and low n-3 test meals strengthened the difference between HTG and HC groups.

Previous studies suggested that increasing consumption of n-3 PUFAs could improve lipid metabolism both in the fasting and postprandial states [20], however in our study, the postprandial lipid response was not different between the high and low n-3 test meals, suggesting that n-3 PUFA needs more than 8 hours to exert these beneficial effects and the fatty acids content of background diet maybe more effective than the type of fatty acids in test meal to influence the postprandial response [21]. Furthermore, the pure fat test with high n-3 PUFAs also can not acutely improve postprandial lipid response in men with metabolic syndrome [22]. Although differences in postprandial responses were not observed between the meals, the pattern of TG response was different in HTG group and HC group. The peak point of the blood TG concentration was 4 h postmeal after eating the high n-3 meals but 6 h after the low n-3 meal in HTG group. The prolonged elevation in TG concentration may reflect the prolonged either slower absorption or to increased lipolysis. Shah et al. [6] also pointed that test meals rich in DHA and EPA (the representative of n-3 PUFAs) may reduce the TG response, but the difference was not significant between DHA and EPA and linoleic acid (the representative of n-6 PUFAs). Given the TC, HDL, ApoA, ApoB is an acknowledge marker of lipid metabolism in the fasting state, in our research the concentration of these were also measured in postprandial samples. But all these markers did not show any marked change over time after meals (data not show), which is accordance with the previous studies [23,24], suggesting that the postprandial rate of their catabolism and synthesis was slow. So measuring these markers will not add new information for postprandial response.

Subjects with fasting hypertriglyceridemia usually have elevated and prolonged postprandial lipid response [4]. In the present study it is apparent that HTG subjects tended to have greater postprandial increase in TG levels relative to healthy controls, which is in good agreement with most studies [25-27]. Furthermore the difference in the magnitude of postprandial TG response was strengthened by low n-3 test meal. After both test meals maximal change and iAUC for TG was all higher in HTG group than HC group, the difference were significant after low n-3 test but not after high n-3 test. Hypertriglyceridemia subjects show slower clearance of glucose, larger insulin increases and more extravagant endothelial dysfunction [28]. Although in our study the concentration of glucose, insulin, IL-6, TNFα and ET-1 at each time point was higher NO was lower in HTG group, but the maximal change and iAUC had no significant difference except for iAUC of insulin, IL-6 and diAUC of NO after low n-3 test meals.

Postprandial period is regarded as pro-inflammatory state, indicated by elevated levels of inflammatory proteins such as IL-6 and TNFα. There is evidence that n-3 fatty acids are anti-inflammatory, but n-6 fatty acids are pro-inflammatory [9,29]. However our results suggest that the ratio of n-6 and n-3 in test meal does not acutely influence inflammatory status in HTC and HC subjects. The magnitude of the postprandial increase in plasma IL-6 and TNFα was similar after high n-3 and low n-3 meals in both groups. Earlier studies have demonstrated that IL-6 increases from morning to night irrespective of food intake [30-32]. So the increase of IL-6 levels in our research maybe confounded because of blood drawing procedure [33]. Plasma TNFα concentration increased in parallel with plasma IL-6 concentration in current study, which could be partly explained by the elevation of the number of monocytes expressing TNFα within visceral adipose tissue [34,35]. Other studies in obesity and diabetes men, TNFα concentrations were unchanged or decreased [36,37]. Possible reasons for conflicting results could be the differences in population in study and composition of the test meal.

The postprandial state, characterized by elevations in TG, is a proinflammatory situation contributing to endothelial dysfunction that is considered the earliest stage of atherosclerotic [38]. NO and ET-1 are major vasodilator and vasoconstrictor endothelium-derived substances respectively, and their role and reciprocal interactions in endothelial function have been extensively accepted. In our research postprandial endothelial function impaired with decreasing of NO and increasing of ET-1 after test meals, which are consistent with many previous studies [39,40]. It is well known that intake of n-3 fatty acids is inversely associated with biomarkers of inflammation and endothelial activation [40,41]. In our current research high n-3 test meal does not improve the postprandial endothelial disfunction in HTC and HC subjects. It is apparent that HTG subjects tended to have greater postprandial decrease in NO levels compared to healthy controls, which further proved that transient increase in TG increase the risk of atherosclerosis. The disordered postprandial metabolism of triglyceride-riched lipoproteins may play an atherogenic role by inducing endothelial dysfunction [42].

## Conclusion

In conclusion, the present results indicated that the ratio of n-6 and n-3 can not acutely influence the postprandial lipid, glucose and insulin metabolism, inflammatory response and endothelial function, but the low n-3 test meal can strengthen the difference between HTG and HC groups.

## Abbreviations

PUFAs: Polyunsaturated fatty acids; TG: Triglycerides; TC: Total cholesterol; TNFα: Tumor necrosis factor alpha; IL-6: Interleukin-6; NO: Nitric oxide; ET-1: Endothelin-1; HC: Healthy control; HTG: Hypertriglyceridemia; TRL: Triglyceride-rich lipoprotein; SFAs: Saturated fatty acids; MUFAs: Monounsaturated fatty acids; iAUC: The incremental areas under the postprandial curve; diAUC: The decremental areas under the postprandial curve AUC; ANOVA: Analysis of variance.

## Competing interests

The authors have declared no competing interests.

## Authors’ contributions

The work was carried out in collaboration between all authors. ZXS and GJS defined the research theme and drafted the manuscript; ZXS, LGY and GFS performed the experiments, acquisition of data, analysis and interpretation of data. GFS and HXL carried out all the biochemical analyses and helped in the writing of the manuscript. All authors read and approved the final manuscript.
